# Supplementary material for: The Determinants of Vaccine Literacy in the Italian Population: Results from the Health Literacy Survey 2019
Source: Int J Environ Res Public Health. 2022 Apr 7;19(8):4429. doi: 10.3390/ijerph19084429 (PMC9029177; doi:10.3390/ijerph19084429)
Supplement: Supplementary file 1 [file ijerph-19-04429-s001.zip › ijerph-1640737-supplementary.pdf]

Study population			Find				Understand				Judge				Decide			
			Very difficult	Difficult	Easy	Very Easy	Very difficult	Difficult	Easy	Very Easy	Very difficult	Difficult	Easy	Very Easy	Very difficult	Difficult	Easy	Very Easy
Total (n=3,500)																		
Sex	n	%																
Male	1685	48.14	4.0	28.7	52.4	15.0	3.4	18.7	53.3	24.6	5.3	30.0	50.0	14.7	3.5	18.3	53.8	24.5
Female	1815	51.86	5.2	28.7	53.7	12.5	3.8	21.6	54.1	20.5	4.7	26.9	53.4	14.9	3.8	21.1	55.0	20.1
Age class																		
18-29	468	13.37	3.8	30.0	47.2	19.0	2.6	16.2	46.7	34.6	4.2	30.6	44.6	20.5	2.5	19.1	51.9	26.5
30-44	826	23.60	4.6	28.1	50.6	16.7	4.3	22.7	46.9	26.1	5.8	31.5	43.7	19.0	4.3	21.4	49.0	25.3
45-64	1,254	35.83	5.5	29.3	53.1	12.1	4.2	20.9	53.8	21.1	5.0	29.0	51.5	14.5	4.2	21.6	52.8	21.4
65+	952	27.20	3.7	27.7	57.9	10.7	2.9	18.9	63.0	15.2	4.7	23.9	62.4	9.0	2.9	16.3	62.3	18.6
Education																		
Low	1,470	42.00	5.1	30.1	54.2	10.6	4.0	23.4	57.2	15.4	4.9	27.2	58.0	10.0	3.7	20.0	59.1	17.2
Medium	1,299	37.11	4.3	28.2	52.4	15.1	3.3	19.3	52.2	25.2	5.3	29.8	48.6	16.4	3.2	21.1	51.1	24.6
High	731	20.89	4.0	26.7	52.0	17.3	3.6	15.3	49.3	31.8	4.6	28.5	44.9	22.0	4.4	16.8	50.6	28.2
Geographic area																		
North-West	939	26.83	4.0	26.7	55.9	13.5	4.1	19.1	51.8	25.0	2.5	19.5	54.6	23.4	2.5	19.5	54.6	23.4
North-East	710	20.29	4.5	27.0	54.3	14.3	2.6	19.5	56.5	21.5	3.5	17.1	53.5	25.9	3.5	17.1	53.5	25.9
Center	681	19.46	4.5	27.0	54.8	13.7	3.1	17.3	55.6	24.0	2.0	18.7	57.0	22.3	2.0	18.7	57.0	22.3
South and islands	1,170	33.43	5.2	32.2	49.1	13.5	4.2	23.2	52.4	20.2	5.7	22.2	53.2	18.9	5.7	22.2	53.2	18.9
Deprivation																		
No	1,478	44.63	2.5	22.6	57.8	17.1	1.9	13.2	58.1	26.8	2.7	22.6	56.4	18.3	1.9	14.5	57.2	26.4
low	539	16.27	3.6	27.8	51.9	16.7	3.2	19.6	52.5	24.7	4.0	28.7	50.9	16.5	2.5	19.4	54.7	23.4
medium	743	22.43	5.8	32.2	52.0	10.0	4.2	24.6	52.8	18.5	5.2	33.4	49.9	11.5	5.1	24.5	52.5	18.0
high	552	16.67	9.8	39.9	43.4	7.0	8.5	32.8	44.3	14.4	11.1	37.0	44.2	7.8	8.5	27.4	49.0	15.2
HL-47 quartile																		
First	896	25.60	13.6	56.8	27.7	1.8	11.5	48.7	34.7	5.2	15.1	61.2	22.4	1.4	10.6	44.9	38.0	6.6
Second	854	24.40	3.8	34.3	54.5	7.4	1.9	21.7	57.4	19.0	3.3	35.3	53.4	8.1	3.3	21.9	57.9	17.0
Third	875	25.00	0.6	19.8	65.6	14.1	0.8	8.7	63.8	26.7	1.2	15.3	67.2	16.3	0.5	10.7	61.0	27.9
Fourth	875	25.00	0.0	3.1	65.2	31.7	0.1	0.9	59.6	39.4	0.1	1.2	65.0	33.8	0.1	0.9	61.3	37.7

Figure S1. Percentage distribution of HL-VAC score level by participant characteristics and HL-VAC process subsections, corresponding to the four HL-VAC items.
